# Necrosis of the Lower Jaw

**Published:** 1855-07

**Authors:** W. G. Smull

**Affiliations:** Baltimore


### ARTICLE VI.

### *Necrosis of the Lower Jaw.*

The following interesting communication has been received by the editors from Dr. W. G. Smull, of Eutaw-st. We call special attention to it as the record of a well observed case of a very important disease, which, in its early stages, is by no means easy of diagnosis. Eds.

Messrs. Editors:

As instances of necrosis of the entire lower jaw are comparatively rare, and as such cases pertain more immediately to your specialty, I have taken the liberty to transcribe the notes of the following case. The progress of the case never led me to doubt the patient's final recovery, until the singular accident mentioned brought it to a speedy and fatal termination.

I hope the hurry with which these notes were for the most part taken and made, without any expectation of publication, will be sufficient apology for their imperfection.

Charles N., aged 10, came under my care on October 9th, 1854. He looks scrofulous, muscles flabby, complexion white and colorless; temperament lymphatic, habits ordinarily active, parents healthy. His health has been good until within the last four months. His mother knows of no cause that could have impaired it. She mentioned that he has been in the habit of bathing his head and face with cold water when over-heated; on one occasion he suffered pain in the right side of his face and over the brow after bathing. This pain has recurred, at times, ever since. Has never taken mercury to salivation, or been confined to his bed from sickness. Four months ago, a circumscribed swelling appeared over the lower maxilla, on the right side, midway between the symphysis and angle of the jaw. This rapidly increased in size and became hard and painful; it was pronounced to be osteo-sarcoma, by a surgeon of this city. The swelling involves the whole right side of the face, from the angle of the eye to the ear, and from the zygoma to a point about two inches below the rim of the jaw. At the point where the swelling first began, a fistulous opening has taken place; on introducing the probe into this orifice, it passed upward and backward without touching the bone; its withdrawal was followed by a fetid sanious discharge, characteristic of necrosis. The teeth appear sound and firm. He complains only of the pain over the brow, mentioned above. He has become weak since the opening occurred, which was about four days ago. His appetite is impaired, spirits depressed. Tongue natural, pulse 100, rather feeble. Organs appear to act normally, though feebly. He is directed to use the most nutritious food, to take out-door exercise, to apply a poultice over the entire tumor, and to take a mixture of bichloride of mercury, cinchona and conium.

Oct. 20th. The swelling has somewhat subsided—another abscess has formed and discharged, leaving a sinus; both now discharging freely. Appetite and strength much improved; ordered, that the sinuses should be injected with Labarraque's solution, chlor. soda.

Oct. 23d. Had the teeth examined by Dr. Wm. Stiles, dentist, in Lexington street, who found the second bicuspid of the right side loose. On attempting to extract it, the molars moved, showing that the alveolar process was separated. He then dissected the gum from the teeth and alveolar process, and with a slight elevation of the forceps brought away two and a half inches of sequestrum with three teeth inserted; a fair view was then had of the bone, it was black and jagged, the permanent teeth were seen in their sockets, imperfectly developed, but loose and black; these were then extracted with considerable difficulty—during the operation the hemorrhage was profuse; directed the whole exposed surface of the bone to have antiseptic and styptic wash applied to it constantly, medicines repeated.

Nov. 1st. Appearance of erysipelas at the lower edge of the tumor. Face much swollen and very painful. The bone is so much exposed, that it can be seen when he opens his mouth. Sinuses have healed. To apply a lotion of opium and sup. acet. plumbi to the whole surface; to take a saline aperient.

Nov. 17th. Swelling subsided—no signs of erysipelas left. The sequestrum is loose at the point where the teeth were extracted. Could not remove it with the forceps. His general condition is not changed—appetite variable. To take the syr. phytolaccæ comp. (or poke root,) with iij grs. iod. potassii three times a day.

Nov. 26th. Patient extracted the large piece of sequestrum last night with his fingers. Examination shows the entire *depth* of the bone is lost at that point. The entire row of front teeth to the second bicuspid on the left side, with their articulations, can be moved with a little effort. In fact, the alveolar process of the whole jaw appears movable. His appetite has improved with the change of medicines. To apply diluted nitric acid to the exposed bone.

Dec. 4th. An abscess has broken over the ramus about two inches above the angle on the right side; face very red and painful; appears to suffer great constitutional irritation. Ordered poultices, saline aperient.

Dec. 5th. Much worse. Slept none last night. Erysipelas has appeared over the whole affected part. Chin much disfigured by swelling. Lips œdematous. Can with difficulty get fluids down the throat. To apply evaporating lotion over the whole surface. To take sulph. morphia, $\frac{1}{8}$ grain, every hour, till sleep is procured.

Dec. 8th. Much improved. Erysipelas disappeared.

Dec. 19th. Several of the front teeth have come away. On turning down the lip the denuded and blackened bone can be seen as far as the first bicuspid on the left side. Removed several pieces of sequestrum.

Jan. 5th. Extracted the entire angle of the jaw of the right side. The exfoliation took place near the articulation with the temporal bone. The sequestrum contained the remnant of the socket of the second molar. No hemorrhage of consequence followed its extraction.

Feb. 1st. The patient at present is much deformed; the chin almost meets the chest; the effusion in the cellular tissue is very great. Callus appears to be forming rapidly to supply the place of the lost bone; the entire chin is loose and moves from its bed at every movement of the jaw. The exfoliation will probably take place at the second bicuspid on the left side. The swelling extends toward the ramus on that side.

Feb. 20th. The cavity left by the exfoliation has been entirely filled by callus. The chin can almost be extracted. Allowed it to remain to prevent the contraction that would ensue from its removal. The disease has extended to the same height on the left that it reached on the opposite side; the bone is painful on pressure, and the swelling indicates the extent of the disease. The patient feels stronger than when first placed under treatment. Appetite unimpaired; says it is not satisfied by taking fluids. No change of treatment.

March 14th. Called to patient at 9, P. M. Found that he had had a frightful hemorrhage and was still bleeding from the mouth. Could not estimate the amount of blood lost before I arrived. The hemorrhage was found to proceed from the right facial artery. Pressure upon the artery increased it, showing that the coats of the artery were cut through by the rough edge of the necrosed bone. Folds of lint saturated with mur. tr. ferri, were placed between the artery and bone and immediately stopped the bleeding. Pulse 150, hardly distinguishable. Allowed a small quantity of wine. To be kept perfectly quiet.

March 16th. At 1½, A. M., patient had another hemorrhage to the amount of ℥ xv. Found him almost insensible. Surface of body bathed in cold perspiration. Could not distinguish pulse at the wrist. Breathing quick and labored. Unable to speak. Used the same means as before with the same success. 2½, P. M., died.

It should be mentioned that the best surgical advice was consulted after the first hemorrhage, as to the propriety of tying the carotid. The facial and external carotid were so much thrown out of their relations by the diseased tissues, that it would have been impracticable to attempt to tie them, and it was thought safer to run the risk of the patient bleeding to death than to attempt to tie the common carotid in the state of extreme prostration in which the hemorrhage left him.

It is to be regretted that an autopsy was not allowed, as the maxilla presented the disease in its various stages of incipient necrosis, exfoliation and formation of callus. The extent to which the coats of the vessels were involved would have been another interesting subject of investigation.

Very Respectfully, Yours,

W. G. SMULL.

*Baltimore, June,* 1855.

